# Comparative Evolutionary Patterns of *Burkholderia cenocepacia* and *B. multivorans* During Chronic Co-infection of a Cystic Fibrosis Patient Lung

**DOI:** 10.3389/fmicb.2020.574626

**Published:** 2020-09-25

**Authors:** A. Amir Hassan, Sandra C. dos Santos, Vaughn S. Cooper, Isabel Sá-Correia

**Affiliations:** ^1^iBB - Institute for Bioengineering and Biosciences, Instituto Superior Técnico, Universidade de Lisboa, Lisbon, Portugal; ^2^Department of Bioengineering, Instituto Superior Técnico, Universidade de Lisboa, Lisbon, Portugal; ^3^Department of Microbiology and Molecular Genetics, University of Pittsburgh School of Medicine, Pittsburgh, PA, United States

**Keywords:** *Burkholderia cepacia* complex, cystic fibrosis, chronic pulmonary infections, within-host evolution, comparative genomic analysis, *B. cenocepacia*, *B. multivorans*

## Abstract

During chronic respiratory infections of cystic fibrosis (CF) patients, bacteria adaptively evolve in response to the nutritional and immune environment as well as influence other infecting microbes. The present study was designed to gain insights into the genetic mechanisms underlying adaptation and diversification by the two most prevalent pathogenic species of the *Burkholderia cepacia* complex (Bcc), *B. cenocepacia* and *B. multivorans.* Herein, we study the evolution of both of these species during coinfection of a CF patient for 4.4 years using genome sequences of 9 *B. multivorans* and 11 *B. cenocepacia.* This co-infection spanned at least 3 years following initial infection by *B. multivorans* and ultimately ended in the patient’s death by cepacia syndrome. Both species acquired several mutations with accumulation rates of 2.08 (*B. cenocepacia*) and 2.27 (*B. multivorans*) SNPs/year. Many of the mutated genes are associated with oxidative stress response, transition metal metabolism, defense mechanisms against antibiotics, and other metabolic alterations consistent with the idea that positive selection might be driven by the action of the host immune system, antibiotic therapy and low oxygen and iron concentrations. Two orthologous genes shared by *B. cenocepacia* and *B. multivorans* were found to be under strong selection and accumulated mutations associated with lineage diversification. One gene encodes a nucleotide sugar dehydratase involved in lipopolysaccharide O-antigen (OAg) biosynthesis (*wbiI*). The other gene encodes a putative two-component regulatory sensor kinase protein required to sense and adapt to oxidative- and heavy metal- inducing stresses. This study contributes to understanding of shared and species-specific evolutionary patterns of *B*. *cenocepacia* and *B*. *multivorans* evolving in the same CF lung environment.

## Introduction

Chronic pulmonary infections are considered the leading cause for morbidity and premature death of patients at risk, as cystic fibrosis (CF) patients and immunocompromised individuals (Lipuma, 2010). Several opportunistic pathogens may populate the CF airways and the heterogeneous microbial populations usually present are difficult to eradicate (Lipuma, 2010). Among the bacterial pathogens, *Burkholderia cepacia* complex (Bcc) species are particularly feared by CF patients (Mahenthiralingam et al., 2005). Currently, the Bcc includes 24 closely related bacterial species (De Smet et al., 2015; Depoorter et al., 2016; Ong et al., 2016; Bach et al., 2017; Weber and King, 2017; Martina et al., 2018). Pulmonary infection with Bcc bacteria can involve a single species or co-infection with more than one Bcc species and, besides interpatient transmission, these bacteria can cause chronic infections and a marked decline of lung functions and decreased life expectancy. In certain cases, some Bcc species can lead to a lethal uncontrolled clinical deterioration with septicemia and necrotizing pneumonia (the “cepacia syndrome”) (Mahenthiralingam et al., 2005).

During chronic infection by Bcc bacteria, the original infecting strain(s) establishes a population that evolves and diversifies into genetically and phenotypically distinct lineages. These lineages are thought to acquire multiple adaptations to the CF lung environment (Coutinho et al., 2011; Lieberman et al., 2011, 2014; Madeira et al., 2011, 2013; Mira et al., 2011; Silva et al., 2011, 2016; Moreira et al., 2014, 2017; Maldonado et al., 2016; Cabral et al., 2017; Hassan et al., 2017, 2019a; Lee et al., 2017; Nunvar et al., 2017). Over the last decade, several virulence factors and adaptive traits have been identified in Bcc species (Mahenthiralingam et al., 2005; Drevinek and Mahenthiralingam, 2010; Loutet and Valvano, 2010; Zlosnik and Speert, 2010; Madeira et al., 2011, 2013; Mira et al., 2011; Zlosnik et al., 2011, 2014; Sousa et al., 2017; Hassan et al., 2019a). Several studies have used genome-wide methods to identify genetic changes during long term infection by *B*. *dolosa* (Lieberman et al., 2011, 2014), *B*. *multivorans* (Silva et al., 2016; Diaz Caballero et al., 2018) and *B*. *cenocepacia* (Lee et al., 2017; Nunvar et al., 2017) within individual hosts. For example, a retrospective study involving 112 sequential *B. dolosa* isolates from 14 CF patients, identified several mutated genes involved in genetic variation within individual patients, in particular genes required for expression of surface polysaccharides, lipopolysaccharide O-antigen (OAg) biosynthesis, outer membrane components, iron scavenging, and antibiotic resistance (Lieberman et al., 2011). A subsequent study using the same sequential *B. dolosa* isolates suggested parallel bacterial evolution where different lineages may coexist for many years within a patient and identified candidate pathogenicity genes (Lieberman et al., 2014). Another comparative genomic study including 22 isolates of *B. multivorans* recovered over 20 years from CF patients also found parallel adaptive variations resulting from host related selection pressures in additional genes involved in the above referred functional categories (Silva et al., 2016). A more recent comparative genomic analysis including 32 clonal variants of *B. cenocepacia* obtained from 8 CF patients (Nunvar et al., 2017) revealed that, in addition to the aforementioned parallel mutations in gene functions previously described for *B*. *dolosa* and *B*. *multivorans* (Lieberman et al., 2011, 2014; Silva et al., 2016), genes related to transition metal metabolism are hotspots for nucleotide polymorphism (Nunvar et al., 2017). Another comparative study involving 215 genomes from serial *B. cenocepacia* isolates obtained from 16 CF patients during a 20 year-period also supports the above mentioned evolutionary trajectories during chronic Bcc infections and the well-established diverse-community model (Lee et al., 2017). This publication also reported the complete loss of chromosome III resulting in genome-size reduction as an adaptive trait of *B. cenocepacia* (Lee et al., 2017), consistent with a previously reported rare *in vivo* loss of the same mega-plasmid among *B. cenocepacia* clonal variants emerging during chronic infection (Moreira et al., 2017). Recently, a population genomic study focused on 111 *B. multivorans* isolates obtained from a Canadian CF patient showed potential parallel pathoadaptation involving genes associated with resistance toward multiple classes of antibiotics (Diaz Caballero et al., 2018).

Although the above genomic analyses have established the field of *Burkholderia* evolution during chronic infection of the CF airways, the comparison of the evolutionary patterns of strains of different species co-infecting the same CF patient is lacking. This was the objective of the present study focused on the most prevalent Bcc species among the CF community worldwide: *B*. *cenocepacia* and *B. multivorans* (Mahenthiralingam et al., 2005; Drevinek and Mahenthiralingam, 2010; Lipuma, 2010). Although *B. cenocepacia* has been identified as the most frequent species among the Bcc bacteria causing infections, with high potential for inter-patient transmission (Mahenthiralingam et al., 2005; Drevinek and Mahenthiralingam, 2010), in several countries *B. multivorans* has replaced *B. cenocepacia* as most frequent (Lipuma, 2010). Given that the CF environment is characterized by high pro-inflammatory cytokine levels, high antibiotic concentrations, high levels of oxidative stress and low oxygen concentration (Moriarty et al., 2007; Palmer et al., 2007; Reid et al., 2007; Williams et al., 2007), we hypothesize that the adaptive evolution of each Bcc species may vary in the same CF patient’ lung-environment. This work was designed to compare the genome sequences of *B. cenocepacia* and *B. multivorans* clonal variants co-inhabiting the same host-selective environment to identify species-specific and shared evolutionary patterns. A retrospective study on twenty clonal variants derived from two ancestor strains (9 *B. multivorans* isolates and 11 *B. cenocepacia* isolates) was performed. They were sequentially retrieved from the same CF patient over a period of 4.4 years, from the onset of infection with *B. multivorans* followed by co-infection with *B. cenocepacia* until the patient’s death from cepacia syndrome (Correia et al., 2008; Coutinho et al., 2011; Hassan et al., 2019a). These isolates were obtained in the major Portuguese CF treatment Center at Hospital de Santa Maria during consultation routines and pulmonary exacerbations that compelled the patient to hospitalization and intravenous therapy with gentamicin and ceftazidime (Correia et al., 2008; Coutinho et al., 2011). The design and successful execution of the present study provided relevant and useful information to contribute to a better understanding of the common and the specific evolutionary patterns occurring in *B*. *cenocepacia* and *B*. *multivorans* under selection in the same CF lung environment and host immune system during a co-infection that ultimately led to the cepacia syndrome.

## Materials and Methods

### Bcc Bacterial Isolates and Growth Conditions

Eleven *B. cenocepacia* (*recA* lineage IIIA) sequential isolates and nine *B. multivorans* sequential isolates obtained from the same cystic fibrosis patient (patient J) (Cunha et al., 2003; Coutinho et al., 2011; Hassan et al., 2019a) were examined in this study. These isolates were collected during routine monitoring at Hospital de Santa Maria (HSM), Centro Hospitalar Lisboa Norte (CHLN) EPE, Lisbon, Portugal, from the sputum of a chronically infected CF patient who was under surveillance from February 1998 to July 2002 (Cunha et al., 2003; Correia et al., 2008; Coutinho et al., 2011; Hassan et al., 2019a; Table 1). These isolates were obtained from the onset of the Bcc infection until the patient’s death with cepacia syndrome after 4.4 years of Bcc infection and were selected at random among the colonies isolated in selective *Burkholderia cepacia* Selectatab medium at the Hospital, at a specific date of isolation. Bacterial cultures are stored at −80°C in 1:1 (v/v) glycerol. Bacteria were grown in Lysogeny broth (LB; Conda, Pronadisa) at 37°C with shaking at 250 rpm or in LB agar plates.

**TABLE 1:** *Burkholderia cepacia* complex isolates examined, ordered based on the isolation date.

Bcc isolate	Isolation date	Bcc species
IST419	26 Feb 1998	*B. multivorans*
IST424	4 Jun 1998	*B. multivorans*
IST439	30 Jan 1999	*B. cenocepacia recA* lineage IIIA
IST453	19 Jul 1999	*B. multivorans*
IST455A/IST455B	1 Feb 2000	*B. multivorans*
IST461	4 Apr 2000	*B. multivorans*
IST495A/IST495B	29 May 2001	*B. multivorans*
IST4103	24 Jul 2001	*B. cenocepacia recA* lineage IIIA
IST4110	25 Sep 2001	*B. cenocepacia recA* lineage IIIA
IST4112	11 Oct 2001	*B. cenocepacia recA* lineage IIIA
IST4113	6 Nov 2001	*B. cenocepacia recA* lineage IIIA
IST4119	22 Jan 2002	*B. multivorans*
IST4116A/IST4116B	11 Feb 2002	*B. cenocepacia recA* lineage IIIA
IST4131	26 Feb 2002	*B. cenocepacia recA* lineage IIIA
IST4129	26 Mar 2002	*B. cenocepacia recA* lineage IIIA
IST4130	14 May 2002	*B. cenocepacia recA* lineage IIIA
IST4134	2 Jul 2002	*B. cenocepacia recA* lineage IIIA

*They were obtained from a cystic fibrosis patient and their genomes were sequenced during this study.*

### Genomic DNA Sequencing, Assembly and Annotation

For genomic DNA extraction, bacterial cultures were prepared by suspending isolated colonies from LB agar plates in 3 mL LB broth, followed by overnight growth at 37°C with shaking at 250 rpm. Genomic DNA was extracted and purified using a DNeasy Blood and Tissue kit (Qiagen, Germany) according to manufacturer instructions. DNA concentration and purity were assessed using a Nanodrop ND-1000 spectrophotometer.

Genomic DNA samples of all the studied clonal variants were processed according to Illumina’s instructions for generating paired-end libraries. In brief, *Burkholderia cenocepacia* IST439, IST4113, IST4129 and IST4134 were sequenced using a whole-genome shotgun sequencing strategy and Illumina Genome Analyser sequencing technology at CD Genomics (New York, NY, United States), generating short 100-bp paired-end reads with high coverage (∼300x), details of this protocol attached as Supplementary Materials. All of the *B. multivorans* clonal variants were sequenced on Illumina NextSeq 500 platform at the University of Pittsburgh, Pittsburgh, PA, United States; isolates of *B. cenocepacia* IST4103, IST4110, IST4112, IST4116A, IST4116B, IST4131 and IST4130 were sequenced using the 151-bp paired-end Illumina HiSeq platform at the University of New Hampshire Hubbard Center for Genomic Studies, after library preparation using a modified Illumina Nextera protocol designed for inexpensive library preparation of microbial genomes (Baym et al., 2015). Raw fastQ reads were analyzed using fastQC, which revealed that all isolates were sequenced at sufficient depth to accurately identify single nucleotide polymorphisms and indel mutations. Raw fastq paired-end files were processed for removal of Illumina adapters, trimming, and quality-based filtering using Trimmomatic v0.32 (Bolger et al., 2014).

*Burkholderia multivorans* IST419 and *B. cenocepacia* IST439, used as ancestor reference strains, were sequenced for a second time to generate a high-quality contiguous assembly. *B. multivorans* IST419 was likewise processed using Illumina NextSeq 500 platform producing a second sets of pair-ended reads, while *B. cenocepacia* IST439 was re-sequenced to generate a complete assembly by using a combination of single molecule, real-time (SMRT) Pacific Biosciences – PacBio reads and Illumina 100-bp paired-end reads (protocol described in Supplementary Materials). We used the hierarchical genome-assembly process workflow (HGAP3) to generate a completed assembly of *B. cenocepacia* IST439 and polished our assembly using the Quiver algorithm (Chin et al., 2013).

The trimmed reads of all the studied clonal variants (except *B. cenocepacia* IST439) were *de novo* assembled using VelvetOptimiser v2.2.4 (Zerbino and Birney, 2008; Zerbino, 2010) and SPAdes v3.11.1-linux (Bankevich et al., 2012) with automated optimization of the assembly parameters. The quality of the assemblies obtained was estimated by Quast v2.3 (Gurevich et al., 2013) and the best assembled outputs were automatically improved using Pilon v1.22 (Walker et al., 2014). The three trimmed data sets (two paired-end libraries and one mate-pair library) were used for scaffolding by SSPACE-standard v3.0 (Boetzer et al., 2011) followed by an automated improvement using Pilon v1.22 to gain the final draft genome sequences.

With draft genome sequences, contigs/scaffolds were reordered using MAUVE Contig Mover (Darling et al., 2010). All assembled-drafted genomes were reordered versus *B. cenocepacia* J2315 and *B. multivorans* ATCC_17616. We then annotated the completed assembly of *B. cenocepacia* IST439 and the remaining draft genome sequences with the prokaryotic genome annotation tool prokka (v1.11) with a local, customized *Burkholderia* database of closely related Bcc strains (*B. cenocepacia* H111, *B. cenocepacia* J2315, *B. cenocepacia* ST32, *Burkholderia cenocepacia* VC1254, *B. dolosa* AU0158, *B. multivorans* ATCC_BAA-247, and *B. multivorans* ATCC 17616) (Seemann, 2014). BLAST Ring Image Generator (BRIG) was used to generate visual genome comparisons with the reference genomes of *B. cenocepacia* H111, *B. cenocepacia* J2315, *B. cenocepacia* ST32, *B. multivorans* ATCC_BAA-247, and *B. multivorans* ATCC 17616 (Alikhan et al., 2011). For enriched-functional annotation, eggNOG-mapper v4.5.1 was additionally applied for both strains (*B. cenocepacia* IST439 and *B. multivorans* IST419) that were used as references for the variant calling (Huerta-Cepas et al., 2017). This COG mapping was used to count the CDS per COG category for each species.

### Variant Calling and SNP/INDEL Detections

Trimmed paired-end reads were mapped against the ancestor reference complete genome of *B. cenocepacia* IST439 (for all *B. cenocepacia* clonal variants) and/or against the ancestor reference draft genome sequence of *B. multivorans* IST419 for the corresponding clonal variants using BWA-MEM packages of Burrows-Wheeler Aligner (BWA v.0.7.10) (Li and Durbin, 2010) and NovoAlign^1^. Variants [Single nucleotide polymorphisms (SNPs) and insertion-deletion mutations (INDELs)] were called as described previously by using two independent standard variant calling pipelines; GATK and SAMtools/BCFtools toolbox (Li et al., 2009; Van der Auwera et al., 2013; Dillon et al., 2015, 2017). A brief description of this approach is included in Supplementary Materials.

To perform a quality control of the results obtained by the above reported method, a parallel comparison was performed using Breseq (Barrick et al., 2014), and similar predictions were found in the case of base-substitutions, with some discrepancies in indels. All putative SNPs/INDELs were then manually inspected and evaluated using the Integrative Genomics Viewer – IGV (Robinson et al., 2011; Thorvaldsdottir et al., 2013), and discarded if the BWA and Novoalign-produced alignments did not provide enough confidence (poor coverage, as described in the Supplementary Materials). Thus, we are confident that nearly all base-substitution and indels identified in this study were genuine events that arose during the *in vivo* evolutionary process. Finally, Functional annotation of the called variants was performed by using SnpEff v3.1 (Cingolani et al., 2012) with manual BLAST verification against the NCBI Microbes genome database.

### Assessment of Population Structure and Phylogeny

*In silico* whole genome multi-locus sequence typing (wgMLST) (Jolley and Maiden, 2010) was performed to confirm the isolate sequence type (ST) and the clonality of all the isolates examined.

To obtain a comprehensive phylogeny of the studied clonal variants, the SNP calls and their matrices were automatically processed by using a set of algorithms previously described (Kaas et al., 2014) via the online web server CSI phylogeny v1.4^2^ with default settings; min depth at SNP position – 10x, relative depth at SNP position – 10%, minimum distance between SNPs – 10 bp, min SNP quality – 30, and min read mapping quality – 25 (Kaas et al., 2014). Phylogenetic trees were then constructed using 70 and 110 variant positions among *B. cenocepacia* and *B. multivorans* clonal variants, respectively, by using FastTree (Price et al., 2010).

Seven *B. cenocepacia recA* lineage IIIA, two *B. multivorans*, and one *B. dolosa* deposited-reference genomes in *Burkholderia* genome database^3^ were used to describe the phylogenetic relationship of the studied clinical isolates among the Bcc bacteria (Supplementary Table 1). Whole genome average nucleotide identity (ANI) was assessed by using fastANI script (Jain et al., 2018).

### Genomic Features and Structural Analysis of the Ancestor *B. cenocepacia* and *B. multivorans* Strains Genomes

*Burkholderia cenocepacia* IST439 and *B. multivorans* IST419 were considered the ancestor reference strains and used to identify subsequent evolved mutations. Reported genomic islands (GIs: BcenGIs and GiST32-s) (Holden et al., 2009; Graindorge et al., 2012; Nunvar et al., 2017) were searched for to identify their presence in *B. cenocepacia* IST439 and *B. multivorans* IST419 genomes using basic local alignment search tool – BLAST (Altschul et al., 1990; Zhang et al., 2000).

The presence of Putative GIs was also predicted using IslandViewer 4 (Bertelli et al., 2017), unique GIs were manually confirmed and curated in the genome sequences of *B. cenocepacia* IST439 and *B. multivorans* IST419. For this confirmation, it was considered that they are unique continuous DNA regions absent in the available genome sequences of the published epidemic clones and longer than 10 kbp, flanked, at both sides, by homologous regions (especially those inserted immediately downstream of tRNA), and have lower (or higher) GC content compared with the rest of the genome (Guo et al., 2017; Nunvar et al., 2017). The absence of DNA regions homologous to these unique GIs in the genomes of epidemic clones – *B. cenocepacia* ET12 and ST32 (Holden et al., 2009; Nunvar et al., 2017) and *B. multivorans* ATCC_17616 and ATCC_BAA-247 (Komatsu et al., 2003; Johnson et al., 2015; Johnson et al., 2016) – was confirmed and visualized by Progressive MAUVE whole genome pairwise alignment and ACT/Artemis (Carver et al., 2005; Darling et al., 2010). Large DNA regions that respected those criteria but were not predicted by IslandViewer, were also manually identified and considered genuine-unique GIs.

The assembled genomes of IST419 and IST439 were also examined to identify acquired antimicrobial resistance –AMR – genes through different databases ResFinder 3.2 (Zankari, 2014) and CARD (Jia et al., 2017) and virulence factors via VFanalyzer from VF-database (Liu et al., 2019). All the pathoadaptive associated genes obtained *in silico* were confirmed by using ABRicate version 0.5^4^.

### Ethics Statement

Studies involving the clinical isolates compared in this study were approved by Centro Hospitalar Lisboa Norte (CHLN)’ ethics committee and the anonymity of the patient is preserved. Informed consent for the use of these isolates in research was obtained from the patient and/or the legal guardians. The patient’s isolates and clinical data was collected as part of the epidemiological survey of Bcc bacteria involved in pulmonary infections among the CF patients receiving care followed at Hospital de Santa Maria (Cunha et al., 2003; Correia et al., 2008).

### Data and Nucleotide Sequence Accession Numbers

DNA sequence reads for all isolates obtained by Illumina sequencing are available at the EMBL’s European Nucleotide Archive (ENA) under accession number PRJEB20052 and PRJEB35836. The fully assembled and annotated genome of *B. cenocepacia* IST439 obtained by PacBio Sequencing is available at ERZ1345975, Supplementary Table 1. The other drafted and annotated genomes of *B. cenocepacia* and *B. multivorans* clonal variants obtained by Illumina Sequencing in the present study are available at Supplementary Table 1.

## Results

### Genomic Analysis of *B. cenocepacia* and *B. multivorans* Ancestor Strains

The first isolates obtained from patient J, *B. cenocepacia* IST439 and *B. multivorans* IST419, were used as the ancestor reference strains for comparative genomic analysis. The genome of *B. cenocepacia* IST439 was sequenced using a combination of PacBio and Illumina sequences, yielding an assembly of three scaffolds corresponding to the three expected replicons (Holden et al., 2009) with a total genome size of 7.63 Mb (Supplementary Table 2). Plasmid pBCJ2315, present in the genome of *B. cenocepacia* J2315 strain (Holden et al., 2009), was not found in *B*. *cenocepacia* IST439 genome. The combination of two Illumina sequencing rounds of *B. multivorans* IST419 allowed the assembly of 76 scaffolds with a predicted genome size of ∼ 6.48 Mb (Supplementary Table 2) corresponding to the three expected replicons (Komatsu et al., 2003; Holden et al., 2009).

The genome sequences of the ancestor reference strains, *B. cenocepacia* IST439 and *B. multivorans* IST419, were screened for the presence of previously reported Genomic Islands (GIs), BcenGIs and GiST32-s (Holden et al., 2009; Graindorge et al., 2012; Nunvar et al., 2017). Most of these metabolic GIs were absent from both genomes but resistance GIs (in chromosome II) and pathogenic GI (Prophage BcepMu in chromosome III) were found (Supplementary Table 3). These shared GIs contain genes associated with arsenic resistance, antibiotic resistance, inorganic-ion and sulfate transporter, and stress response (Baldwin et al., 2004; Holden et al., 2009) and the *B. cenocepacia* low-oxygen-activated *lxa* locus (Sass et al., 2013). Another shared pathogenic GI, BcepMu prophage, contains genes involved in replication, regulation and pathogenesis (Summer et al., 2004) reported to be overexpressed in response to high doses of exogenous reactive oxygen species (ROS) (Peeters et al., 2010). Fourteen unique GIs were predicted in *B. cenocepacia* IST439 genome (MLST profile ST218) and termed BcenST218_GI1 to BcenST218_GI14 (Supplementary Figure 1 – panel A and Supplementary Table 4) while 17 unique GIs were detected in *B. multivorans* IST419 (MLST profile ST836) and termed BmST836_GI1 to BmST836_GI17 (Supplementary Figure 1 – panel B and Supplementary Table 5). All these unique GIs that were not shared by the *B. cenocepacia* and *B. multivorans* strains include multiple phage-related genes and/or genes associated with metabolism, replication, regulation and pathogenesis, and hypothetical membrane proteins (Supplementary Tables 4, 5).

*Burkholderia cenocepacia* IST439 (ST-218) was found to be closely related to the German *B. cenocepacia* H111 isolate [ST1506, 99.2% average nucleotide identity (ANI)] obtained from sputum of a CF patient (Carlier et al., 2014) and more distantly to the worldwide ET-12 epidemic clones J2315 (99.0% ANI), BC7 (98.9% ANI), and K56-2 (99.0% ANI) (Holden et al., 2009; Varga et al., 2013) (Supplementary Figure 2 and Supplementary Table 1). *B. multivorans* IST419 (ST-836) is more related to the Belgian clone *B. multivorans* ATCC_BAA-247 (ST650, 99.0% ANI) retrieved from sputum of a CF patient (Johnson et al., 2015; Johnson et al., 2016) than to the U.S. soil reference isolate *B. multivorans* ATCC_17616 (ST21, 96.9% ANI) (Komatsu et al., 2003) (Supplementary Figure 2 and Supplementary Table 1).

### Comparative Analysis of the Genome Sequences of *B. cenocepacia* and *B. multivorans* Sequential Clonal Isolates

**FIGURE 1:**
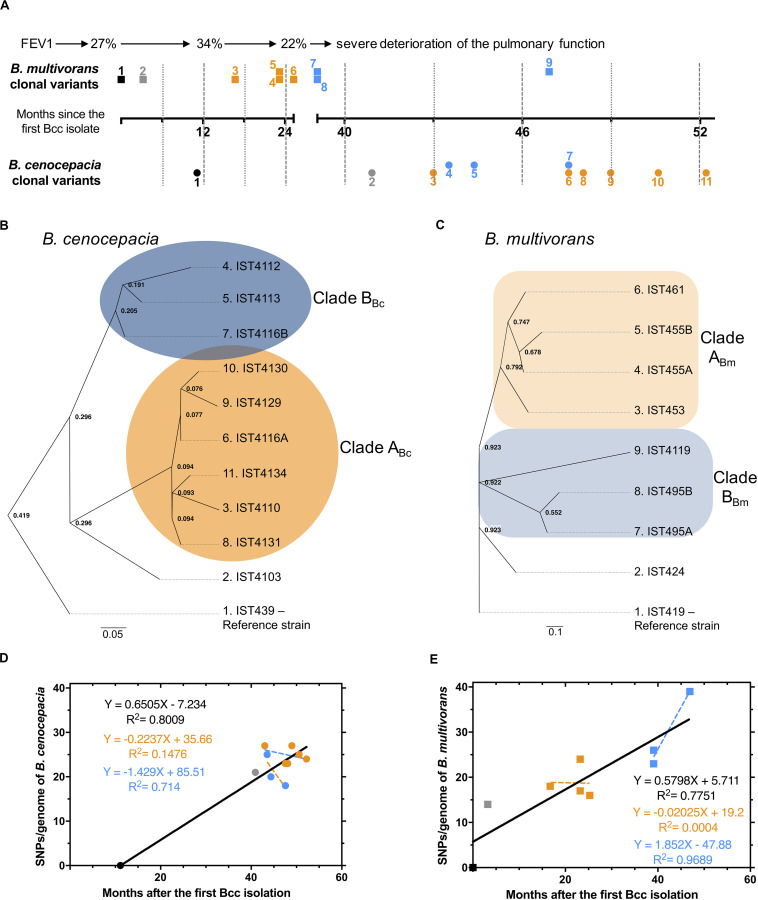
**(A)** Schematic representation of the *B. cenocepacia* (•) and *B. multivorans* (■) isolates examined with information on lung function (Forced Expiratory Volume in the first second, FEV1%). Time points of isolation, in months after the first Bcc isolate was retrieved, are shown; the numerical order [also used in the phylogenetic trees of *B. cenocepacia* **(B)** and *B. multivorans* **(C)**] and the two colors used correlate with the two clades described for *B. cenocepacia* and *B. multivorans* clonal variants. **(D and E)** represent SNP accumulation during chronic infection and the relation between the time elapsed from the first Bcc isolation to the point of isolation of each isolate. A linear fit with a slope was plotted. Bc and Bm indicates *B. cenocepacia* and *B. multivorans*, respectively.

The genomes of the eleven *B. cenocepacia* isolates and of the nine *B. multivorans* isolates were examined to identify mutations arising in the same CF patient during 4.4 years of chronic airway infection (Figure 1 – panel A). These isolates were sequentially obtained from the onset of the Bcc infection with the *B. multivorans* followed by a co-infection with *B*. *cenocepacia* for at least three years until the patient’s death (Correia et al., 2008; Coutinho et al., 2011; Hassan et al., 2019a). The genome of two colonies of different morphotypes (assigned as A and B) that were isolated at the same isolation date for each species [Figure 1, (Coutinho et al., 2011; Hassan et al., 2019a)] were also compared.

All isolates from the same species shared the same MLST type, confirming their clonal relationship (Supplementary Table 2). Putative mutations were identified relative to the genomes of *B. cenocepacia* IST439 or *B*. *multivorans* IST419 (Figure 1 – panels B and C), revealing 70 or 110 variant positions among *B. cenocepacia* and *B. multivorans* clonal variants, respectively. These positions were used to evaluate their phylogenetic relationships (Figure 1), which suggest that the two Bcc populations originated from a single colonization event followed by subsequent diversification leading to two different sub-populations (clades A_*Bc*_ and A_*Bm*_; B_*Bc*_ and B_*Bm*_) for each Bcc species (Figure 1 – panels B and C – orange and blue for clade A_*Bc*_, A_*Bm*_ and B_*Bc*_, B_*Bm*_ – respectively). The two *B. cenocepacia* clades (clades A_*Bc*_ and B_*Bc*_) were detected emerging during a common infection period while *B. multivorans* clade B_*Bm*_ was detected subsequently to clade A_*Bm*_ (Figure 1 – panels A and B). The genetic differentiation among isolates did not always follow the chronology of isolation. For instance, isolates IST4110 and IST4134 are closely related although they were isolated years apart, indicating long standing coexistence of the two *B. cenocepacia* subpopulations (Figure 1 – panels A and B). Moreover, the sole pair of *B. cenocepacia* isolates IST4116A/B obtained at the same isolation date, three years after the isolation of the first *B. cenocepacia* isolate IST439, belongs to distinct lineages (Figure 1 – panels A and B)

### Evolutionary Dynamics of *B. cenocepacia* and *B. multivorans* Co-infecting Populations Inside the CF Lung

A total of 63 single-nucleotide polymorphisms (SNPs) and 9 insertions-deletions (INDELs) were identified among *B. cenocepacia* isolates, and 97 SNPs and 16 INDELs among *B. multivorans* clonal variants (Supplementary Tables 6, 7). SNP accumulation rates (calculated as linear regression slope, Figure 1 – panels D and E) indicate that the mutation rates for *B. cenocepacia* and *B. multivorans* populations were 2.08 and 2.27 SNPs/year, respectively. Most of the mutations, especially those observed along with the clades’ emergence and shared among multiple isolates, are non-synonymous (Tables 2, 3, Supplementary Tables 6, 7) and affect coding sequences (CDSs). For instance, 22 non-synonymous and 1 synonymous mutations were associated with two clades A_*Bc*_ and B_*Bc*_ that emerged within the *B. cenocepacia* population and 35 non-synonymous and 7 synonymous were related to the two clades A_*Bm*_ and B_*Bm*_ that emerged in the *B. multivorans* population. Such enrichment of non-synonymous mutations is greater than the reported substitution under neutral evolution given the codon usage and GC content of *B. cenocepacia* bacteria (Dillon et al., 2015). These results suggest that positive selection dominated during the evolution of Bcc in the CF patient lung (Tables 2, 3 and Supplementary Figure 3). Similar positive selection has been reported for other Bcc strains evolving in the CF airway, including *B. cenocepacia*, *B. multivorans* and *B. dolosa* species (Lieberman et al., 2011, 2014; Silva et al., 2016; Lee et al., 2017; Nunvar et al., 2017; Diaz Caballero et al., 2018).

**TABLE 2:** Summary of selected non-synonymous mutations found among *B. cenocepacia* clonal variants associated with phylogenetic clades (clades A_*Bc*_ and B_*Bc*_, Figure 1 – panel B, depicted in lighter and darker colors, respectively).

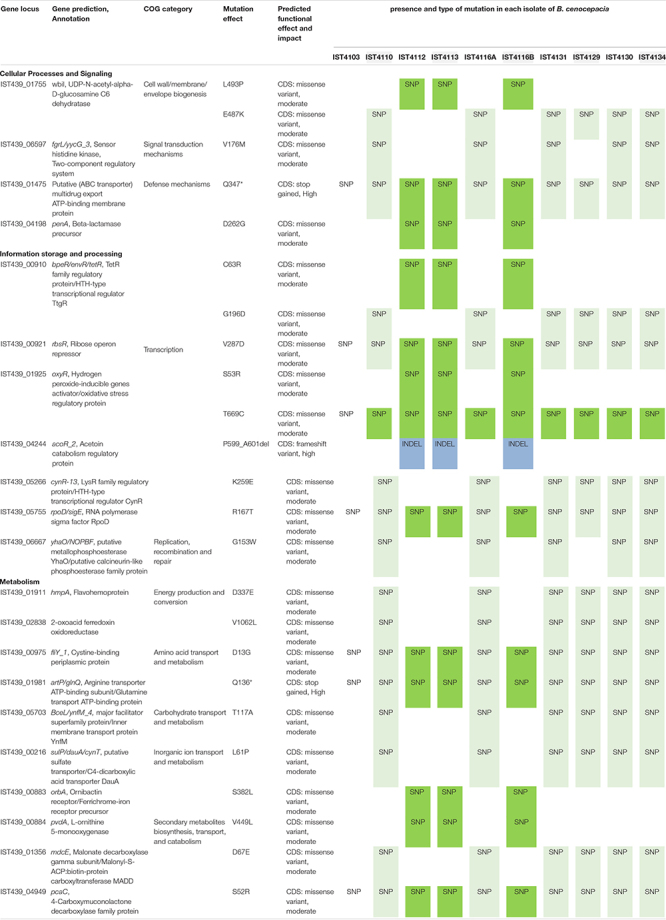

*Gene loci are ordered based on COG annotation and its categories. More details in Supplementary Table 6.*

**TABLE 3:** Summary of selected non-synonymous mutations found among *B. multivorans* clonal variants associated with phylogenetic clades (clades A_*Bm*_ and B_*Bm*_, Figure 1 – panel C, depicted in lighter and darker colors, respectively).

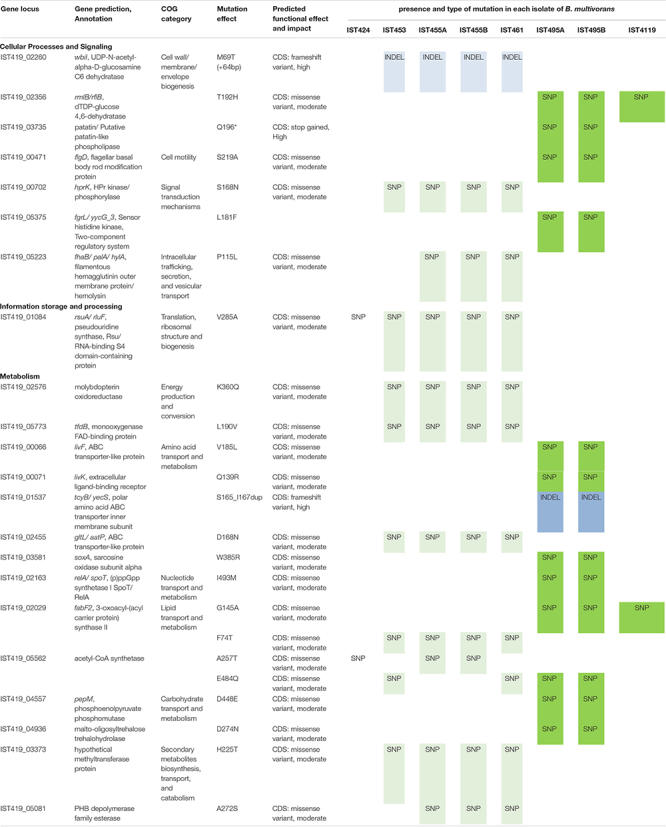

*Gene loci are ordered based on COG annotation and its categories. More details in Supplementary Table 7.*

Consistent with selection acting on *B. cenocepacia* clonal variants of clade B_*Bc*_, only a few genes accumulated mutations that were not also found in the other clade A_*Bc*_ (Table 2 and Supplementary Table 6). For instance, within clade B_*Bc*,_ the gene homologous to the IST439_04198 locus, a *penA* homolog from *B. cenocepacia* J2315 that encodes a putative beta-lactamase, was found to acquire a D262G mutation in clones IST4112, IST4113 and IST4116B. This mutation might be a result of the drastic intravenous therapy with gentamicin and ceftazidime and in agreement with the previously reported high resistance to the beta-lactam antibiotic ceftazidime (Coutinho et al., 2011). Also, among clade B_*Bc*_ variants, one indel was observed (P599_A601deletion) in the IST439_04244 gene, which is homologous to BCAM2211 in *B. cenocepacia* J2315 that is an ortholog of *acoR* from *Bacillus subtilis*. This *acoR* gene encodes a sigma-54 dependent transcriptional regulator that is involved in the regulation of the acetoin catabolic pathway (Huang et al., 1999; Ali et al., 2001). The acetoin catabolic pathway is believed to be a defense mechanism to avoid the lethal acidification produced by CF pathogens during fermentation and anaerobic respiration as a result of the decreased oxygen availability inside the CF lung (Whiteson et al., 2014). Moreover, other two mutations (S382L and V449L) were observed in genes homologous to IST439_00883 and IST439_00884 loci, respectively. These two loci are homologous to *orbA* and *pvdA*, respectively, that encode ornibactin siderophore biosynthesis and transport proteins (Agnoli et al., 2006). A *pvdA* deletion mutant was found to be less virulent than the parent strain in chronic and acute models of respiratory infection (Sokol et al., 1999) and a *B. cenocepacia* K56-2 *orbA* mutant (K56*orbA*:tp, *orbA* allelic exchange mutant) has also an attenuated virulence in the rat chronic respiratory infection model (Sokol et al., 2000). The reported functions of the four genes that were mutated within clade B_*Bc*_ suggest that positive selection might be driven by well-known environmental factors of the CF lung, in particular high antibiotic concentrations, low oxygen concentrations and low iron concentrations. On the other hand, *B. cenocepacia* clonal variants that form clade A_*Bc*_ accumulated mutations found in regulatory and metabolism related genes (Table 2). This suggests that *B. cenocepacia* tend to adapt within the CF airway by remodeling their metabolism during long term infection with periodic diversification as a function of variation within host environments. In *B. multivorans*, most of the mutated genes also affected metabolic functions (energy production and conservation and regulation of the carbohydrate metabolism) (Table 3).

### *Burkholderia cenocepacia* and *B*. *multivorans* Genes Under Convergent Evolution in the CF Lung

The mutated genes during chronic infection belong to different clusters of orthologous groups – COG (Supplementary Figure 3 and Tables 2, 3, Supplementary Tables 6, 7). Genes with well-characterized functions that accumulated non-synonymous mutations (41 and 48 *B. cenocepacia* and *B. multivorans* CDSs, respectively) are schematized in Supplementary Figure 3 and selected mutations are listed in Tables 2, 3.

The genes most likely under selection in the CF airway are those with non-synonymous mutations that become fixed within all subsequent samples. One of these is the *B. cenocepacia* gene linked to defense mechanisms against antibiotic stress (IST439_01475, encoding a putative ABC transporter ATP-binding membrane protein; homologous to BCAL1039 from *B. cenocepacia* J2315) (Table 4). Three other mutations affected genes involved in transcription regulation, encoding the ribose operon repressor RbsR and two sigma factor cytoplasmic proteins homologous to *B. cenocepacia* J2315 RpoC and RpoD proteins. Three mutations were also detected in genes associated with metabolism (BCAL1610 and *glnQ* homologs – encoding periplasmic cysteine-binding protein and glutamine ABC transporter ATP-binding protein – and *pcaC* homolog encoding 4-carboxymuconolactone decarboxylase, involved in the metabolism of amino acids and secondary metabolites, respectively). On the other hand, no single non-synonymous mutation became fixed among *B. multivorans* isolates during the chronic co-infection (Tables 3 and Supplementary Table 7). Although the simultaneous, apparent fixation of seven new mutations over 18 months may seem high, manual inspection of the evidence supporting these mutations indicates they are genuine. It is possible that these mutations may have accumulated in a lineage evolving over a longer period of time prior to the first sampling, when clones like IST439 and more evolved lineages co-occurred.

**TABLE 4:** List of non-synonymous mutations (remarked with √) that became fixed among the clonal variants of *B. cenocepacia* examined.

Gene locus/homologous gene in J2315	Annotation	Mutational effect	COG	IST439	IST4103	IST4110	IST4112	IST4113	IST4116A	IST4116B	IST4131	IST4129	IST4130	IST4134
IST439_01475/BCAL1039 homolog	Putative (ABC transporter) multidrug export ATP-binding/permease membrane protein	Q347*	V		√	√	√	√	√	√	√	√	√	√
IST439_00921/ *rbsR* homolog	Ribose operon repressor	V287D	K		√	√	√	√	√	√	√	√	√	√
IST439_02295/ *rpoC* homolog	DNA-directed RNA polymerase subunit beta	T669C	K		√	√	√	√	√	√	√	√	√	√
IST439_05755/ *rpoD/sigE* homolog	RpoD/SigE, RNA polymerase sigma factor RpoD	R167T	K		√	√	√	√	√	√	√	√	√	√
IST439_00975/BCAL1610 homolog	FliY_1, Cystine-binding periplasmic protein	D13G	E		√	√	√	√	√	√	√	√	√	√
IST439_01981/ *glnQ* homolog	Glutamine transport ATP-binding protein GlnQ/ABC transporter-like protein	Q136*	E		√	√	√	√	√	√	√	√	√	√
IST439_4949/ *pcaC* homolog	PcaC, 4-Carboxymuconolactone decarboxylase family protein	S52R	Q		√	√	√	√	√	√	√	√	√	√

*The only mutations presented are those acquired by CDS associated with well-characterized functions as shown in Supplementary Table 6.*

**FIGURE 2:**
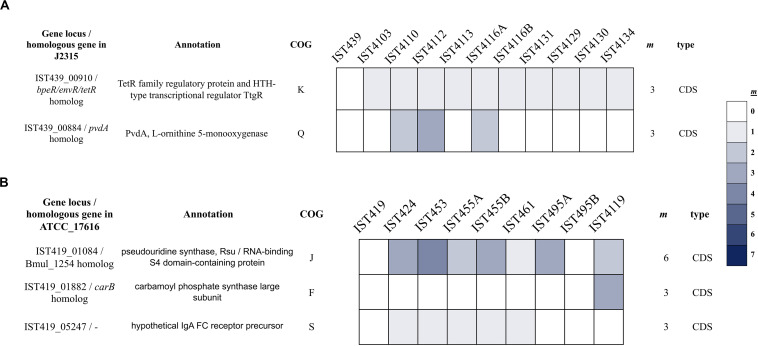
Heat-map of non-synonymous mutation frequencies in coding sequences (CDS) under strong parallelism (that acquired ≥ 3 mutations) among the clonal variants of *B. cenocepacia* **(A)** and *B. multivorans* **(B)**. Homologous genes in *B. cenocepacia* J2315 and *B. multivorans* ATCC_17616 are indicated. Total number of mutations acquired per gene (m) and the type of mutation (coding sequence [CDS]) are presented on the right panel.

Among the mutated genes, two genes in *B. cenocepacia* and three genes in *B. multivorans* acquired multiple independent mutations, which provide strong evidence of selection (Figure 2). Although multiple mutations in a single gene can result from mismapped reads and/or hidden paralogy, the evidence for these mutations is robust following manual inspection. Notably, in *B. cenocepacia*, the gene IST439_00910 that is homologous to *B. cenocepacia* J2315 *bpeR* acquired three different mutations during co-infection; one mutation (G196D) became fixed in clade A_*Bc*_ variants while mutation C63R emerged in clade B_*Bc*_ and one INDEL (K113^∗^ [-57bp]) was only found in the second sequential clonal variant isolated, IST4103, the isolate that does not belong to any of the two defined clades (Figure 1 – panel B). The *bpeR* gene encodes a TetR family regulatory protein which is a conserved transcription regulator across the *Burkholderia* genus (Ramos et al., 2005). This gene was involved in the transcriptional control of multidrug efflux pumps, antibiotic biosynthesis, response to osmotic stress and toxic chemicals, and in pathogenicity in other Gram-negative pathogens (Ramos et al., 2005). The IST439_00910/*bpeR* is also an ortholog to *B. dolosa* AU0158 BDAG_00732 that was found before to acquire several mutations in the promotor region of 112 CF-clinical isolates (Lieberman et al., 2014). Among the three polymorphic genes in *B. multivorans* clonal variants, one polymorphic gene (IST419_01084) accumulated six different mutations and one of these variant positions (V285A) became fixed in the first 6 clonal variants (Figure 2 – panel B). This IST419_01084 gene encodes a putative RNA pseudouridine synthase whose structure contains a small RNA-binding protein domain (S4 domain) that delivers nucleotide-modifying enzymes to RNA, regulating translation through structure specific RNA binding (Aravind and Koonin, 1999; Del Campo et al., 2001).

### Orthologous Genes Experienced Positive Selection in Co-evolved *B. cenocepacia* and *B. multivorans* Populations

**FIGURE 3:**
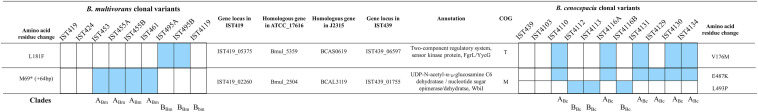
Mutations that were observed in two orthologous genes in the genomes of clonal variants of *B. multivorans* and of *B. cenocepacia* (by chronological order from left to right) are indicated by the amino acid residue change.

Two orthologous genes accumulated mutations related to lineage diversification in both *B. cenocepacia* and *B. multivorans* (Figure 3). These genes are homologs of *B. cenocepacia* J2315 *wbiI* and BCAS0619 and are predicted to encode a nucleotide sugar dehydratase involved in lipopolysaccharide O-antigen (OAg) biosynthesis (Ortega et al., 2005) and a putative two-component regulatory sensor kinase protein, respectively.

Regarding *wbiI*, E487K and L493P mutations were observed in IST439_01755 gene in all *B. cenocepacia* clonal variants forming clades A_*Bc*_ and B_*Bc*_, respectively, while the M69T (+ 64bp) insertion was observed in IST419_02260 gene in all *B. multivorans* clonal variants forming clade A_*Bm*_. Regardless of the Bcc species, all the isolates with a mutated *wbiI* gene expressed a lipopolysaccharide lacking the OAg (Hassan et al., 2019a). Remarkably, this *wbiI* gene was reported before to be under strong selection in *B. cenocepacia*, *B. multivorans* and *B. dolosa* during chronic lung infection (Lieberman et al., 2011, 2014; Silva et al., 2016; Hassan et al., 2017). Concerning BCAS0619, located at the third chromosome, mutation V176M was found within *B. cenocepacia* variants that form clade A_*Bc*_, except for the IST4129 isolate that lacks chromosome III, while IST419_05375 gene has a L181F mutation in two *B. multivorans* variants (IST495A and IST495B).

## Discussion

This study was designed to gain insights into the molecular mechanisms underlying genomic diversification occurring in *B. cenocepacia* and *B. multivorans* strains that co-infected, for a period of at least 3 years, the same cystic fibrosis (CF) patient and to identify shared and relevant pathoadaptive mechanisms. Mutations with accumulation rates of 2.08 (*B. cenocepacia*) and 2.27 (*B. multivorans*) SNPs/year were found during the course of co-infection comparable with those reported for *B. dolosa* [2.1 SNPs/year] (Lieberman et al., 2011, 2014) and *B. multivorans* [2.4 SNPs/year] (Silva et al., 2016) and slightly higher than the mutation rate reported for the Czech epidemic *B. cenocepacia* ST32 strain [average rate 1.66 SNPs/year] (Nunvar et al., 2017). Such mutation rates are also comparable to those occurring in the major CF pathogen *P. aeruginosa* [2.7 SNPs/year] during CF chronic infection (Markussen et al., 2014; Marvig et al., 2015) but below the mutation rates reported for the non-Bcc member and etiological agent of melioidosis, *B. pseudomallei*, obtained from chronically infected CF patients [3.6 SNPs/year] (Viberg et al., 2017). The aforementioned mutation rates are believed to be associated with fast diversification of the infecting bacterial strains (Didelot et al., 2016) diverging into sublineages with their own functional and genomic signatures and rates of adaptation to different CF-lung environments (Markussen et al., 2014; Didelot et al., 2016).

In the co-infecting *B. cenocepacia* and *B. multivorans* populations examined, several genes were found to acquire different mutations either related or unrelated to lineage diversification. Remarkably, most of these genes have not been previously reported as being involved in parallel evolution in *B. dolosa*, *B. cenocepacia*, and *B. multivorans* (Lieberman et al., 2011, 2014; Silva et al., 2016; Nunvar et al., 2017). Specifically, most of the mutated genes found in this work are involved in cell envelope/wall/membrane biogenesis and in regulatory and metabolic processes, belonging to the functional categories reported before for Bcc strains as being under selective pressure in the CF lungs (Lieberman et al., 2011, 2014; Silva et al., 2016; Nunvar et al., 2017). Mutated genes with fixed non-synonymous variant positions over the entire period of chronic infection were found in the present work among the *B. cenocepacia* clonal variants examined but no single fixed non-synonymous variant position was found for *B. multivorans*. Results suggest that *B. cenocepacia* and *B. multivorans* infection involved periods of diversification dominated by positive selection, as previously suggested by others (Silva et al., 2016; Lee et al., 2017; Nunvar et al., 2017). Although we cannot discard the possibility that the spatiotemporal characteristics of the isolated clonal variants examined in this work are not representative of the population heterogeneity present at each isolation time in the CF airways, the distinct evolutionary patterns here attributed to *B*. *cenocepacia* and *B*. *multivorans* when co-infecting the same CF environment for several years, appear to be consistent and in line with the literature. Population heterogeneity in the CF lungs was observed for both species and different colony morphotypes were isolated at the same isolation dates (Figure 1 – panels A, B and C). However, the very limited number of isolates obtained at the same isolation date and the fact that they were not always isolated randomly but to obtain different colony morphotypes limits our inferences of population dynamics. Nevertheless, heterogeneity within the *B. cenocepacia* population can be seen in the two *B cenocepacia* isolates (IST4116A/B) obtained on the same day, which belong to different lineages (clades A_*Bc*_ and B_*Bc*_).

Among the mutated genes in *B*. *cenocepacia* and *B*. *multivorans* during long-term co-infection of the CF patient are genes encoding oxidative stress regulatory proteins and heavy metal-sensing proteins (Tables 2, 3 and Supplementary Tables 6, 7). Such regulatory proteins (e.g., OxyR and FixK) are involved in bacterial protection against reactive oxygen species (ROS) produced by phagocytic leukocytes (Reyrat et al., 1993; Saito et al., 2003; Bobik et al., 2006; Panmanee et al., 2008). Consistent with this conclusion, the host immune system was proposed to play a fundamental role in driving *B. cenocepacia* and *B. multivorans* evolution during chronic CF infection and genes linked to the oxidative stress responses (homologous to *B. cenocepacia* J2315 *katG*, *yedY*, *moeA1*, *fixL, osmC* and *ompR*) reported to suffer mutation (Silva et al., 2016; Nunvar et al., 2017). This selection driven by the host immune system of genes linked to the oxidative stress and heavy metal-sensing was not however proposed for within-patient evolution of either *B. dolosa* or *P. aeruginosa* (Lieberman et al., 2011, 2014; Winstanley et al., 2016).

Increased resistance to antibiotics is also a well-described evolutionary trait associated with many CF pathogens and genes related with antibiotic resistance have been among the genes mostly reported to be under selective pressure (Jeannot et al., 2008; Lieberman et al., 2011, 2014; Silva et al., 2016; Winstanley et al., 2016; Nunvar et al., 2017). Consistent with those reports, some of the mutated genes found in the present study in *B*. *cenocepacia* and *B*. *multivorans* are involved in defense mechanisms and their regulation, as it is the case of genes encoding PenA, EmrA, ABC-2 type transporter, and DNA gyrase proteins (Tables 2, 3 and Supplementary Tables 6, 7).

Another commonly reported evolved trait during chronic infection is mutation altering genes of the OAg biosynthetic cluster (Lieberman et al., 2011, 2014; Maldonado et al., 2016; Silva et al., 2016; Hassan et al., 2017; Nunvar et al., 2017). Longitudinal comparative genomic studies of Bcc isolates from CF patients with chronic lung infection revealed the conversion from smooth (in the early isolates) to rough LPS with no OAg at the late-stages of infections by *B. cenocepacia* and *B. multivorans* (Silva et al., 2016; Hassan et al., 2017). Remarkably, an extensive study focused on *B. dolosa* during chronic lung infections has shown that late isolates produce an LPS exhibiting the OAg that was absent from the LPS of the initial infecting isolate (Lieberman et al., 2011). The phenotypic switch of OAg presence/absence was recently examined among CF isolates of several Bcc species. In the most prevalent and feared species, *B. cenocepacia* and *B. multivorans*, a marked tendency to lose the OAg along chronic infection was seen, whereas in *B. dolosa* the OAg-chain was absent from the beginning of the 5.5-year infection until the patient death (Hassan et al., 2019a). It remains important to understand why LPS is under selection in *B. cenocepacia* or *B. multivorans*. While the most common explanation invokes selection for immune evasion, it is also possible that LPS modifications are adaptations to biofilm growth (Traverse et al., 2013) or antibiotic resistance (Scribner et al., 2020). Another explanation for loss of OAg is that it increases Bcc survival in phagocytic eukaryotic cells such as amoebae, epithelial cells and human macrophages (Saldias et al., 2009; Saldias and Valvano, 2009; Kotrange et al., 2011; Maldonado et al., 2016). It is also known that the OAg absence leads to increased internalization of *B*. *cenocepacia* into macrophages upon phagocytosis (Saldias et al., 2009; Kotrange et al., 2011) and to facilitate *B*. *multivorans* growth inside macrophages (Schmerk and Valvano, 2013). Moreover, the OAg presence was found to interfere with the adhesion of *B. cenocepacia* to bronchial epithelial cells (Saldias et al., 2009) although it was reported to increase the ability of *B. cenocepacia* to adhere to atomic force microscope Si_3_N_4_ tip (Hassan et al., 2019b). Collectively, the loss of the OAg seems advantageous for disease development associated with *B. cenocepacia* or *B. multivorans* infections. Different mutations were accumulated in relation to the lineage emergence in the two co-infecting populations in genes IST439_01755 and IST419_02260 (homolog and ortholog to *wbiI*, respectively – Figure 3) that encode a nucleotide sugar dehydratase, previously found to be under parallel evolution in *B. cenocepacia* or in *B. multivorans* (Silva et al., 2016; Hassan et al., 2017). Remarkably, the comparison of the ability of three of the *B. cenocepacia* isolates examined in the present work to subvert the host’s immune function, assessed by internalization assays using human dendritic cells, showed that the late variants, IST4113 and IST4134, were significantly more internalized exhibiting increased survival within dendritic cells than the early isolate IST439 (Cabral et al., 2017) the sole with an LPS with OAg (Hassan et al., 2017). Altogether, mutated genes’ functions involved in the observed adaptive evolution of the two species co-inhabiting the same host-selective environment are consistent with the idea that positive selection in *B. cenocepacia* and *B. multivorans* might be driven by the action of the host immune system.

Among the few mutated regulatory proteins identified in this study is the histidine kinase protein from *B. cenocepacia* IST439_06759 (an *hisS* homolog) and from *B. multivorans* IST419_05375 that enable bacterial cells to sense and adapt to oxidative- and heavy metal- inducing stresses (Wolanin et al., 2002; Skerker et al., 2005). The orthologous genes encoding the histidine kinase protein acquired two mutations, V176M and L181F, in the two co-infecting *B. cenocepacia* and *B. multivorans* populations, respectively (Figure 3). This is the first time that this transcription regulator is reported to undergo adaptive evolution during CF patient infection. Signal transduction histidine kinase-related proteins are very important regulatory proteins involved in signaling cascades contributing to several simultaneous coordinated responses (Wolanin et al., 2002; Skerker et al., 2005) to, for example, osmoregulation (Tomomori et al., 1999) and chemotaxis (Bilwes et al., 1999) in bacterial pathogens.

*Burkholderia cenocepacia* genes encoding oxidative stress- and heavy metal-sensing- proteins were hypothesized as being the targets of convergent evolution underlying higher affinity to intra-macrophage persistence and the development of the fatal cepacia syndrome (Lee et al., 2017; Nunvar et al., 2017). Although the mechanisms underlying the fatal cepacia syndrome still remains largely unknown, the results obtained in this study are in line with those described by Nunvar et al. (2017) who hypothesized that macrophages, whose bactericidal activity relies on hydrogen peroxide and copper, are putative key players behind the development of the cepacia syndrome.

In summary, the comparative genomic analysis performed in this work identified genes which are under strong positive selection in the most prevalent and most feared Bcc species that co-infected the lung of a CF patient who died from cepacia syndrome. Among them, polymorphic genes and others involved mainly in regulatory and metabolic functions were sorted out. Since cystic fibrosis is a genetic disorder associated with inflammation, sub-optimal antioxidant protection and the continuous use of antimicrobial therapy, all resulting in marked oxidative stress (Galli et al., 2012), it is likely that in the evolved strains several physiological processes might be activated via the global stress responses which in turn can modulate the course of infection. Global stress responses were found to be under parallel evolution in experimentally evolved *P. aeruginosa* biofilms (Winstanley et al., 2016) and were recently observed during chronic lung infections, irrespective to the presence/absence of other members of the CF microbiome (Vandeplassche et al., 2019). Given that both co-infecting Bcc species are known for their ability of intracellular survival inside macrophages (Saldias et al., 2009; Saldias and Valvano, 2009; Kotrange et al., 2011; Schmerk and Valvano, 2013), the mutated genes associated with cell envelope biogenesis, including those responsible for lipopolysaccharide OAg biosynthesis, could promote Bcc persistence through intracellular survival.

This study provides useful information to better understand the shared and species-specific evolutionary patterns of *B. cenocepacia* and *B. multivorans* evolving in the same CF lung environment during co-infection. The two orthologous genes shared by *B. cenocepacia* and *B. multivorans* that accumulate mutations associated with lineage diversification strongly suggest that two traits, lipopolysaccharide O-antigen (OAg) biosynthesis and the sensing and response to oxidative- and heavy metal- induced stresses, are biological processes under strong selection during evolution in the CF lung environment.

## Data Availability Statement

The datasets presented in this study can be found in online repositories. The names of the repository/repositories and accession number(s) can be found in the article/Supplementary Material.

## Author Contributions

SS and VC carried out the first comparative analysis of *B. cenocepacia* genome sequences and AH completed this analysis and performed the genome analysis of *B. multivorans* isolates in collaboration with VC. IS-C designed and coordinated the study and wrote the manuscript with contributions from AH and VC. All authors contributed to the article and approved the submitted version.

## Conflict of Interest

The authors declare that the research was conducted in the absence of any commercial or financial relationships that could be construed as a potential conflict of interest.

## Footnotes

1 www.novocraft.com

2 https://cge.cbs.dtu.dk/services/CSIPhylogeny/

3 https://www.burkholderia.com/

4 https://github.com/tseemann/abricate

## Supplementary Material

Supplementary Figure 1 Genomic islands **(A)** BcenST281_GIs and **(B)** BmST836_GIs in the reference strains, *B. cenocepacia* IST439 and *B. multivorans* IST419, respectively. The inner circles denote genomes of the reference isolates and the corresponding clonal variants, ordered as indicated in the legend; Bc – *B. cenocepacia* isolates and Bm – *B. multivorans* strains. Blue and orange colors denote different clades as indicated in Figure 1. All clonal variants were chronologically ordered based on the isolation date. Visualizations were carried out by BRIG (Alikhan et al., 2011). For more details, see Supplementary Tables 4, 5.

Supplementary Figure 2 Phylogenetic tree based on the comparison of the whole-genome of *B. cenocepacia* and *B. multivorans* clonal variants and other sequenced strains available at *Burkholderia* genome database (Supplementary Table 1). ANI percentages are also presented relative to each studied reference strain, *B. cenocepacia* IST439 and *B. multivorans* IST419.

Supplementary Figure S3 3D plot of non-synonymous mutations observed in *B. cenocepacia* **(A)** and *B. multivorans* **(B)** clonal variants during the chronic co-infection. All mutated genes of well-characterized functions are depicted as green oval shapes (with SNPs) and/or blue shapes (with INDELs). The number of mutated genes per each COG category is illustrated above each corresponding shape as a ratio (number of genes per isolate/number of all mutated genes in the entire population for this particular COG). Accumulation of small green shapes indicates the number of SNP mutations that was observed per isolate. For a detailed list of mutations, see Supplementary Tables 6, 7 for *B. cenocepacia* and *B. multivorans*, respectively. Abbreviation letters representing the COGs are detailed in the bottom panel.

Supplementary Table S1 List of the whole genome sequences of Bcc isolates used in the present study for comparative genomic analyses. The isolation year and country are indicated as well as the multi-locus sequence typing (MLST). CS – cepacia syndrome.

Supplementary Table S2 Metadata information of the studied clonal variants sequentially ordered based on the isolation date; details on genomic assembly and functional annotation are shown as well as the *in silico* MLST profiles.

Supplementary Table S3 Published genomic islands (Holden et al., 2009; Graindorge et al., 2012; Nunvar et al., 2017) present in *B. cenocepacia* J2315 and ST32 and their eventual presence in *B. cenocepacia* IST439 and *B. multivorans* IST419.

Supplementary Table S4 BcenST218_GIs as Genomic islands of *B. cenocepacia* IST439 and their presence in *B. multivorans* IST419, *B. cenocepacia* J2315 and ST32, *B. multivorans* ATCC_17616 and ATCC_BAA-247.

Supplementary Table S5 BmST836_GIs as Genomic islands of *B. multivorans* IST419 and their presence in *B. cenocepacia* IST439, *B. cenocepacia* J2315 and ST32, *B. multivorans* ATCC_17616 and ATCC_BAA-247.

Supplementary Table S6 List of mutations found among *B. cenocepacia* clonal variants. All mutated genes were grouped to predicted gene functions (COG). Isolates are chronologically ordered as indicated in Figure 1. A – GATK pipeline and B – SAMtools/BCFtools toolbox.

Supplementary Table S7 List of mutations found among *B. multivorans* clonal variants. All mutated genes were grouped to predicted gene functions (COG). Isolates are chronologically ordered as indicated in Figure 1. A – GATK pipeline and B – SAMtools/BCFtools toolbox.
