# Supplementary material for: Comparative Evolutionary Patterns of Burkholderia cenocepacia and B. multivorans During Chronic Co-infection of a Cystic Fibrosis Patient Lung
Source: Front Microbiol. 2020 Sep 25;11:574626. doi: 10.3389/fmicb.2020.574626 (PMC7545829; doi:10.3389/fmicb.2020.574626)
Supplement: Supplementary Figure 1 — Genomic islands (A) BcenST281_GIs and (B) BmST836_GIs in the reference strains, B. cenocepacia IST439 and B. multivorans IST419, respectively. The inner circles denote genomes of the reference isolates and the corresponding clonal variants, ordered as indicated in the legend; Bc – B. cenocepacia isolates and Bm – B. multivorans strains. Blue and orange colors denote different clades as indicated in Figure 1. All clonal variants were chronologically ordered based on the isolation date. Visualizations were carried out by BRIG (Alikhan et al., 2011). For more details, see Supplementary Tables 4, 5. [file Image_1.PDF]

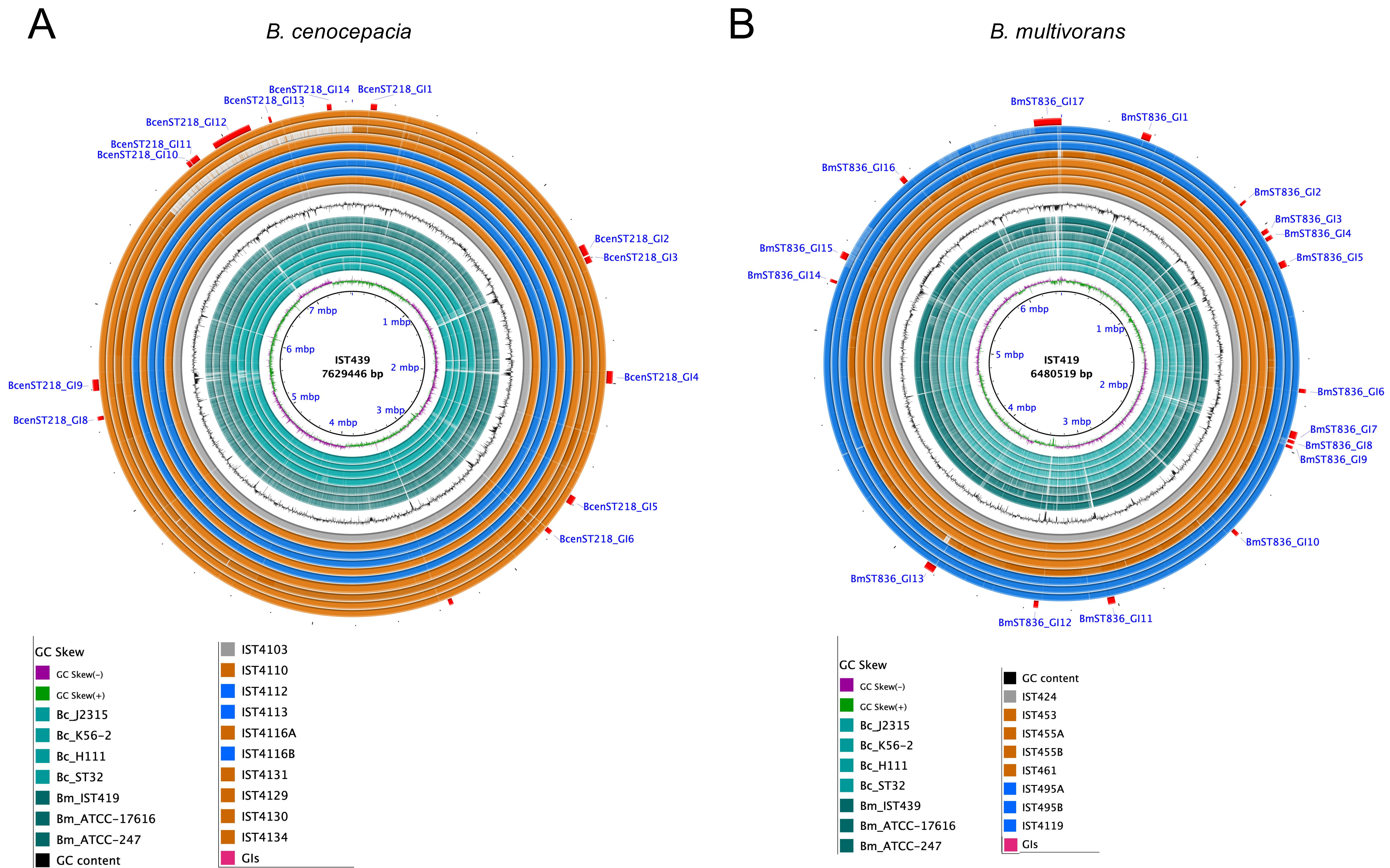
A
B. cenocepacia
BcenST218_GI14
BcenST218_GI1
BcenST218_GI13
BcenST218_GI12
BcenST218_GI11
BcenST218_GI10
BcenST218_GI2
BcenST218_GI3
BcenST218_GI4
BcenST218_GI9
BcenST218_GI8
BcenST218_GI5
BcenST218_GI6
7 mbp
1 mbp
6 mbp
IST439
7629446 bp
2 mbp
5 mbp
3 mbp
4 mbp
GC Skew
GC Skew(-)
GC Skew(+)
Bc_J2315
Bc_K56-2
Bc_H111
Bc_ST32
Bm_IST419
Bm_ATCC-17616
Bm_ATCC-247
GC content
IST4103
IST4110
IST4112
IST4113
IST4116A
IST4116B
IST4131
IST4129
IST4130
IST4134
GIs
B
B. multivorans
BmST836_GI17
BmST836_GI1
BmST836_GI16
BmST836_GI2
BmST836_GI3
BmST836_GI4
BmST836_GI15
BmST836_GI5
BmST836_GI14
BmST836_GI6
BmST836_GI7
BmST836_GI8
BmST836_GI9
BmST836_GI10
BmST836_GI13
BmST836_GI12
BmST836_GI11
6 mbp
1 mbp
5 mbp
IST419
6480519 bp
2 mbp
4 mbp
3 mbp
GC Skew
GC Skew(-)
GC Skew(+)
Bc_J2315
Bc_K56-2
Bc_H111
Bc_ST32
Bm_IST439
Bm_ATCC-17616
Bm_ATCC-247
GC content
IST424
IST453
IST455A
IST455B
IST461
IST495A
IST495B
IST4119
GIs
