# Supplementary figures and images for: Comparative Evolutionary Patterns of Burkholderia cenocepacia and B. multivorans During Chronic Co-infection of a Cystic Fibrosis Patient Lung
Source: Front Microbiol. 2020 Sep 25;11:574626. doi: 10.3389/fmicb.2020.574626 (PMC7545829; doi:10.3389/fmicb.2020.574626)

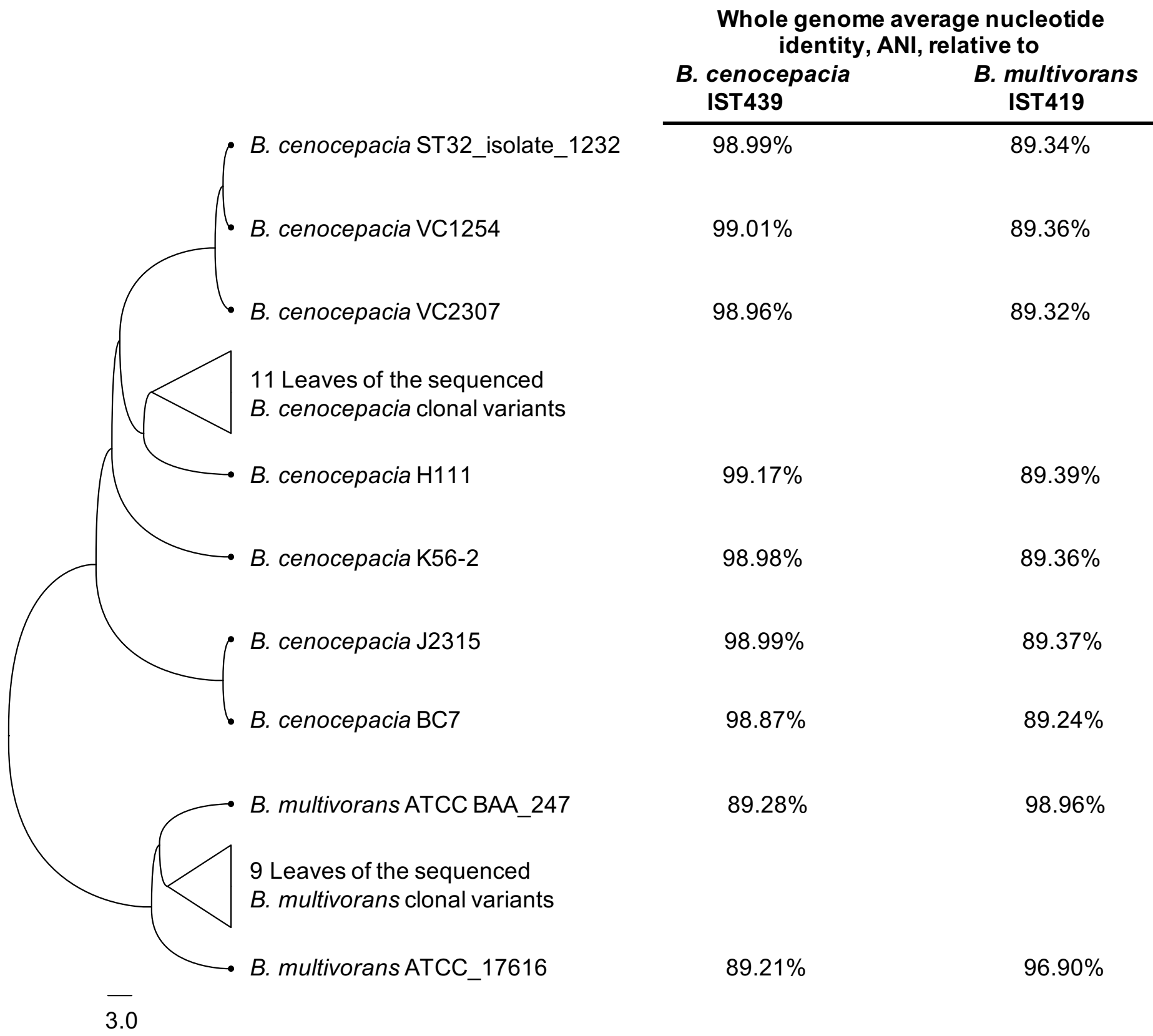

Supplement: Supplementary Figure 2 — Phylogenetic tree based on the comparison of the whole-genome of B. cenocepacia and B. multivorans clonal variants and other sequenced strains available at Burkholderia genome database (Supplementary Table 1). ANI percentages are also presented relative to each studied reference strain, B. cenocepacia IST439 and B. multivorans IST419. [file Image_2.PDF]
