# Supplementary material for: Comparative Evolutionary Patterns of Burkholderia cenocepacia and B. multivorans During Chronic Co-infection of a Cystic Fibrosis Patient Lung
Source: Front Microbiol. 2020 Sep 25;11:574626. doi: 10.3389/fmicb.2020.574626 (PMC7545829; doi:10.3389/fmicb.2020.574626)
Supplement: Supplementary Figure S3 — 3D plot of non-synonymous mutations observed in B. cenocepacia (A) and B. multivorans (B) clonal variants during the chronic co-infection. All mutated genes of well-characterized functions are depicted as green oval shapes (with SNPs) and/or blue shapes (with INDELs). The number of mutated genes per each COG category is illustrated above each corresponding shape as a ratio (number of genes per isolate/number of all mutated genes in the entire population for this particular COG). Accumulation of small green shapes indicates the number of SNP mutations that was observed per isolate. For a detailed list of mutations, see Supplementary Tables 6, 7 for B. cenocepacia and B. multivorans, respectively. Abbreviation letters representing the COGs are detailed in the bottom panel. [file Image_3.PDF]

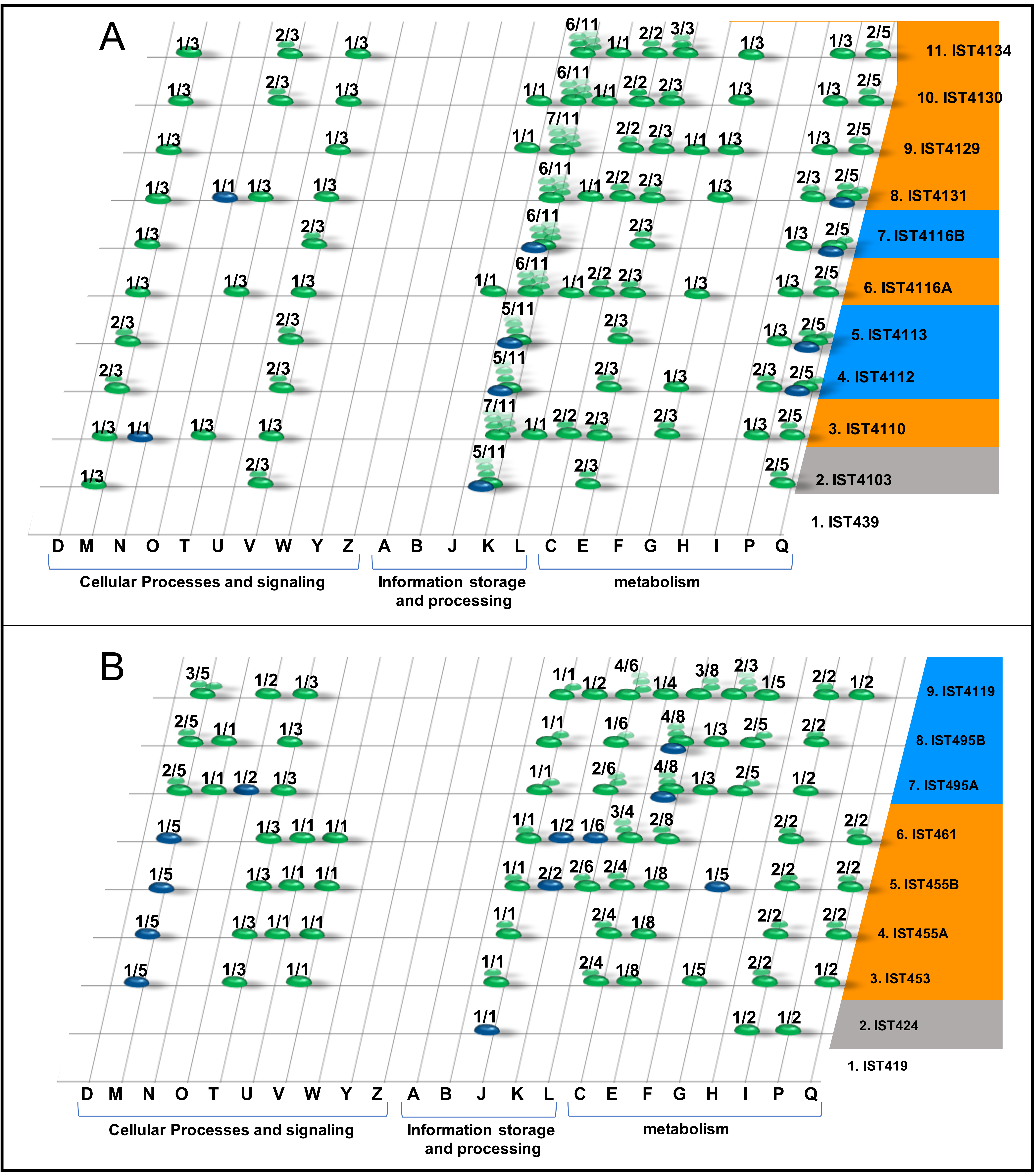

D Cell cycle control and cell division
M Cell wall/membrane/envelope biogenesis
N Cell motility
O Post-translational modification
T Signal transduction mechanisms
U Intracellular trafficking and secretion
V Defense mechanisms
W Extracellular structures
Y Nuclear structure
Z Cytoskeleton
A RNA processing and modification
B Chromatin structures and dynamics
J Translation and ribosomal biogenesis
K Transcription
L Replication and repair system
C Energy production and conversation
E Amino acid metabolism and transport
F Nucleotide metabolism and transport
G Carbohydrate metabolism and transport
H Coenzyme metabolism and transport
I Lipid metabolism and transport
P Inorganic ion metabolism
Q Secondary metabolites biosynthesis, transport and catabolism
