# Supplementary material for: Comparative Evolutionary Patterns of Burkholderia cenocepacia and B. multivorans During Chronic Co-infection of a Cystic Fibrosis Patient Lung
Source: Front Microbiol. 2020 Sep 25;11:574626. doi: 10.3389/fmicb.2020.574626 (PMC7545829; doi:10.3389/fmicb.2020.574626)
Supplement: Supplementary Table S1 — List of the whole genome sequences of Bcc isolates used in the present study for comparative genomic analyses. The isolation year and country are indicated as well as the multi-locus sequence typing (MLST). CS – cepacia syndrome. [file Data_Sheet_2.zip › Table S1.DOCX]

Comparative evolutionary patterns of *Burkholderia cenocepacia* and *B. multivorans* during chronic co-infection of a cystic fibrosis patient lung

A. Amir Hassan^1,2,#^, Sandra C. dos Santos^2^, Vaughn S. Cooper^3^, Isabel Sá-Correia^1,2,^*

^1^ iBB - Institute for Bioengineering and Biosciences, Instituto Superior Técnico, Universidade de Lisboa, Av. Rovisco Pais, 1049-001 Lisbon, Portugal

^2^ Department of Bioengineering, Instituto Superior Técnico, Universidade de Lisboa, Av. Rovisco Pais, 1049-001 Lisbon, Portugal

^3^ Department of Microbiology and Molecular Genetics, University of Pittsburgh School of Medicine, Bridgeside Point II, 450 Technology Drive, Pittsburgh, PA 15219, USA

**^#^ current affiliation:** Protein Research Unit - PRU, Laboratory of Microbiology - LM, Department of Biochemistry and Microbiology, Ghent University, Ghent, Belgium

*** Correspondence:**Professor Isabel Sá-Correia
[isacorreia@tecnico.ulisboa.pt](mailto:isacorreia@tecnico.ulisboa.pt)

**Running title:** Bcc species coevolution in the CF-lung

Keywords: *Burkholderia cepacia* complex, cystic fibrosis, chronic pulmonary infections, within-host evolution, comparative genomic analysis, *B. cenocepacia*, *B. multivorans*

**Table S1.** List of the whole genome sequences of Bcc isolates used in the present study for comparative genomic analyses. The isolation year and country are indicated as well as the multi-locus sequence typing (MLST). CS – cepacia syndrome.

| **Strain** | **Isolation Year** | **Isolation Country** | **Isolation Source** | **MLST sequence type** | **MLST clonal complex** | **Status** | **Accession** | **Reference** |
| --- | --- | --- | --- | --- | --- | --- | --- | --- |
| *Burkholderia cenocepacia IST439* | 1999 | Portugal | CF sputum-CS | ST218 | CC31 | complete | GCA_903231545 | This study |
| *Burkholderia cenocepacia IST4103* | 2001 | Portugal | CF sputum-CS | ST218 | CC31 | draft | GCA_903470625 | This study |
| *Burkholderia cenocepacia IST4110* | 2001 | Portugal | CF sputum-CS | ST218 | CC31 | draft | GCA_903231465 | This study |
| *Burkholderia cenocepacia IST4112* | 2001 | Portugal | CF sputum-CS | ST218 | CC31 | draft | GCA_903231515 | This study |
| *Burkholderia cenocepacia IST4113* | 2001 | Portugal | CF sputum-CS | ST218 | CC31 | draft | GCA_903231535 | This study |
| *Burkholderia cenocepacia IST4116A* | 2002 | Portugal | CF sputum-CS | ST218 | CC31 | draft | GCA_903231445 | This study |
| *Burkholderia cenocepacia IST4116B* | 2002 | Portugal | CF sputum-CS | ST218 | CC31 | draft | GCA_903231525 | This study |
| *Burkholderia cenocepacia IST4131* | 2002 | Portugal | CF sputum-CS | ST218 | CC31 | draft | GCA_903231415 | This study |
| *Burkholderia cenocepacia IST4129* | 2002 | Portugal | CF sputum-CS | ST218 | CC31 | draft | GCA_903231425 | This study |
| *Burkholderia cenocepacia IST4130* | 2002 | Portugal | CF sputum-CS | ST218 | CC31 | draft | GCA_903470695 | This study |
| *Burkholderia cenocepacia IST4134* | 2002 | Portugal | CF sputum-CS | ST218 | CC31 | draft | GCA_903231475 | This study |
| [*Burkholderia cenocepacia J2315*](https://www.ncbi.nlm.nih.gov/genome/475?genome_assembly_id=300159) | 1989 | UK | CF sputum-CS | ST28 | CC31 | complete | [GCA_000009485.1](https://www.ncbi.nlm.nih.gov/assembly/GCA_000009485.1) | (Holden et al., 2009) |
| [*Burkholderia cenocepacia*](https://www.ncbi.nlm.nih.gov/genome/475?genome_assembly_id=260721) *K56-2* | 1999 | Canada | CF sputum-CS | ST227 | CC31 | draft | [GCA_000981305.1](https://www.ncbi.nlm.nih.gov/assembly/GCA_000981305.1) | (Varga et al., 2013) |
| [*Burkholderia cenocepacia*](https://www.ncbi.nlm.nih.gov/genome/475?genome_assembly_id=260721) *BC7* | prior to 1992 | Canada | CF sputum-CS | ST28 | CC31 | draft | [GCA_000333135.2](https://www.ncbi.nlm.nih.gov/assembly/GCA_000333135.2) | (Varga et al., 2013) |
| [*Burkholderia cenocepacia H111*](https://www.ncbi.nlm.nih.gov/genome/475?genome_assembly_id=229618) | 1993 | Germany | CF sputum | ST[1506](https://pubmlst.org/bigsdb?page=profileInfo&db=pubmlst_bcc_seqdef&scheme_id=1&profile_id=1506) | - | complete | [GCA_000236215.4](https://www.ncbi.nlm.nih.gov/assembly/GCA_000236215.4) | (Carlier et al., 2014) |
| [*Burkholderia cenocepacia*](https://www.ncbi.nlm.nih.gov/genome/475?genome_assembly_id=260721) *ST32* | 1997 | Czech Republic | CF sputum-CS | ST32 | CC31 | complete | [GCA_001484665.1](https://www.ncbi.nlm.nih.gov/assembly/GCA_001484665.1) | (Dedeckova et al., 2013) |
| [*Burkholderia cenocepacia*](https://www.ncbi.nlm.nih.gov/genome/475?genome_assembly_id=260721) *VC1254* | 1985 | Canada | CF sputum | ST32 | CC31 | complete | [GCA_001999925.1](https://www.ncbi.nlm.nih.gov/assembly/GCA_001999925.1) | (Lee et al., 2017) |
| [*Burkholderia cenocepacia*](https://www.ncbi.nlm.nih.gov/genome/475?genome_assembly_id=260721) [*VC2307*](https://www.ncbi.nlm.nih.gov/genome/475?genome_assembly_id=307158) | 1987 | Canada | CF sputum | ST210 | CC31 | complete | [GCA_001999805.1](https://www.ncbi.nlm.nih.gov/assembly/GCA_001999805.1) | (Lee et al., 2017) |
| [*Burkholderia dolosa AU0158*](https://www.ncbi.nlm.nih.gov/genome/1401?genome_assembly_id=231416) | - | USA | CF patient | ST472 | - | complete | [GCA_000959505.1](https://www.ncbi.nlm.nih.gov/assembly/GCA_000959505.1) | (Johnson et al., 2015; Johnson et al., 2016) |
| [*Burkholderia multivorans ATCC 17616*](https://www.ncbi.nlm.nih.gov/genome/729?genome_assembly_id=300338) | - | USA | Soil isolate | ST21 | - | complete | [GCA_000018505.1](https://www.ncbi.nlm.nih.gov/assembly/GCA_000018505.1) | (Komatsu et al., 2003) |
| [*Burkholderia multivorans ATCC BAA-247*](https://www.ncbi.nlm.nih.gov/genome/729?genome_assembly_id=228551) | - | Belgium | CF sputum | ST650 | - | complete | [GCA_000959525.1](https://www.ncbi.nlm.nih.gov/assembly/GCA_000959525.1) | (Johnson et al., 2015; Johnson et al., 2016) |
| *Burkholderia multivorans IST419* | 1998 | Portugal | CF sputum-CS | ST836 | - | draft | GCA_903470545 | This study |
| *Burkholderia multivorans IST424* | 1998 | Portugal | CF sputum-CS | ST836 | - | draft | GCA_903470515 | This study |
| *Burkholderia multivorans IST453* | 1999 | Portugal | CF sputum-CS | ST836 | - | draft | GCA_903470585 | This study |
| *Burkholderia multivorans IST455A* | 2000 | Portugal | CF sputum-CS | ST836 | - | draft | GCA_903470555 | This study |
| *Burkholderia multivorans IST455B* | 2000 | Portugal | CF sputum-CS | ST836 | - | draft | GCA_903470535 | This study |
| *Burkholderia multivorans IST461* | 2000 | Portugal | CF sputum-CS | ST836 | - | draft | GCA_903470575 | This study |
| *Burkholderia multivorans IST495A* | 2001 | Portugal | CF sputum-CS | ST836 | - | draft | GCA_903470565 | This study |
| *Burkholderia multivorans IST495B* | 2001 | Portugal | CF sputum-CS | ST836 | - | draft | GCA_903470605 | This study |
| *Burkholderia multivorans IST4119* | 2002 | Portugal | CF sputum-CS | ST836 | - | draft | GCA_903470685 | This study |

Carlier, A., Agnoli, K., Pessi, G., Suppiger, A., Jenul, C., Schmid, N., et al. (2014). Genome Sequence of *Burkholderia cenocepacia* H111, a Cystic Fibrosis Airway Isolate. *Genome Announcements* 2(2). doi: 10.1128/genomeA.00298-14.

Dedeckova, K., Kalferstova, L., Strnad, H., Vavrova, J., and Drevinek, P. (2013). Novel diagnostic PCR assay for *Burkholderia cenocepacia* epidemic strain ST32 and its utility in monitoring infection in cystic fibrosis patients. *Journal of Cystic Fibrosis* 12(5)**,** 475-481. doi: 10.1016/j.jcf.2012.12.007.

Holden, M.T., Seth-Smith, H.M., Crossman, L.C., Sebaihia, M., Bentley, S.D., Cerdeno-Tarraga, A.M., et al. (2009). The genome of *Burkholderia cenocepacia* J2315, an epidemic pathogen of cystic fibrosis patients. *Journal of Bacteriology* 191(1)**,** 261-277. doi: 10.1128/JB.01230-08.

Johnson, S.L., Bishop-Lilly, K.A., Ladner, J.T., Daligault, H.E., Davenport, K.W., Jaissle, J., et al. (2016). Correction for Johnson et al., Complete Genome Sequences for 59 *Burkholderia* Isolates, Both Pathogenic and Near Neighbor. *Genome Announcements* 4(2). doi: 10.1128/genomeA.00313-16.

Johnson, S.L., Bishop-Lilly, K.A., Ladner, J.T., Daligault, H.E., Davenport, K.W., Jaissle, J., et al. (2015). Complete genome sequences for 59 *burkholderia* isolates, both pathogenic and near neighbor. *Genome Announcements* 3(2). doi: 10.1128/genomeA.00159-15.

Komatsu, H., Imura, Y., Ohori, A., Nagata, Y., and Tsuda, M. (2003). Distribution and organization of auxotrophic genes on the multichromosomal genome of *Burkholderia multivorans* ATCC 17616. *Journal of Bacteriology* 185(11)**,** 3333-3343. doi: 10.1128/JB.185.11.3333-3343.2003.

Lee, A.H., Flibotte, S., Sinha, S., Paiero, A., Ehrlich, R.L., Balashov, S., et al. (2017). Phenotypic diversity and genotypic flexibility of *Burkholderia cenocepacia* during long-term chronic infection of cystic fibrosis lungs. *Genome Research* 27(4)**,** 650-662. doi: 10.1101/gr.213363.116.

Varga, J.J., Losada, L., Zelazny, A.M., Kim, M., McCorrison, J., Brinkac, L., et al. (2013). Draft Genome Sequences of *Burkholderia cenocepacia* ET12 Lineage Strains K56-2 and BC7. *Genome Announcements* 1(5). doi: 10.1128/genomeA.00841-13.
