# Supplementary material for: Comparative Evolutionary Patterns of Burkholderia cenocepacia and B. multivorans During Chronic Co-infection of a Cystic Fibrosis Patient Lung
Source: Front Microbiol. 2020 Sep 25;11:574626. doi: 10.3389/fmicb.2020.574626 (PMC7545829; doi:10.3389/fmicb.2020.574626)
Supplement: Supplementary file 5 [file Data_Sheet_1.docx]

Comparative evolutionary patterns of *Burkholderia cenocepacia* and *B. multivorans* during chronic co-infection of a cystic fibrosis patient lung

A. Amir Hassan^1,2,#^, Sandra C. dos Santos^2^, Vaughn S. Cooper^3^, Isabel Sá-Correia^1,2,^*

^1^ iBB - Institute for Bioengineering and Biosciences, Instituto Superior Técnico, Universidade de Lisboa, Lisbon, Portugal

^2^ Department of Bioengineering, Instituto Superior Técnico, Universidade de Lisboa, Lisbon, Portugal

^3^ Department of Microbiology and Molecular Genetics, University of Pittsburgh School of Medicine, Pittsburgh, PA, United States

**^#^ current affiliation:** ProteinResearch Unit - PRU, Laboratory of Microbiology - LM, Department of Biochemistry and Microbiology, Ghent University, Ghent, Belgium

*** Correspondence:**Professor Isabel Sá-Correia
[isacorreia@tecnico.ulisboa.pt](mailto:isacorreia@tecnico.ulisboa.pt)

**Running title:** Bcc species coevolution in the CF-lung

Keywords:*Burkholderiacepacia* complex, cystic fibrosis, chronic pulmonary infections, within-host evolution, comparative genomic analysis, *B. cenocepacia*, *B. multivorans*

**Supplementary notes on Materials and Methods**

### Genomic DNA sequencing, *De novo* assembly, and annotation

**Illumina sequencing by CD Genomics**

We used a whole-genome shotgun sequencing strategy and Illumina Genome Analyser sequencing technology. A 100 bp paired-end run was performed with the strains*Burkholderia cenocepacia* IST439, IST4113, IST4129 and IST4134. Genomic DNA was sheared by a nebulizer to generate DNA fragments for the Illumina Paired-End (PE) Sequencing method. DNA libraries (20 ng/μl) were constructed by ligating the specific oligonucleotides (Illumina adapters) designed for PE sequencing to both ends of DNA fragments with the TA cloning method. The ligated DNA was then size-selected on a 2% agarose gel. DNA fragments of ~ 500 bp were excised from the preparative portion of the gel. DNA was then recovered using a Qiagen gel extraction kit and was PCR amplified to produce the final DNA library. Five picomoles of DNA from each strain were loaded onto two lanes of the sequencing chip, and the clusters were generated on the cluster generation station of the GAIIx using the Illumina cluster generation kit. Bacteriophage X174 DNA was used as a control. In the case of paired-end reads, distinct adaptors from Illumina were ligated to each end with PCR primers that allowed reading of each end as separate runs. The sequencing reaction was run for 100 cycles (tagging, imaging, and cleavage of one terminal base at a time), and four images of each tile on the chip were taken in different wavelengths for exciting each base-specific fluorophore. For paired-end reads, data were collected as two sets of matched 100-bp reads. Reads for each of the indexed samples were then separated using a custom Perl script. Image analysis and base calling were done using the Illumina GA Pipeline software.

**Adapters sequence:**

5' P-GATCGGAAGAGCTCGTATGCCGTCTTCTGCTTG

5' ACACTCTTTCCCTACACGACGCTCTTCCGATCT

**PacBio sequencing by a real-time (SMRT) Pacific Biosciences**

*B. cenocepacia* IST439 was later-sequenced to generate a complete assembly by using a combination of single molecule, real-time (SMRT) Pacific Biosciences – PacBio reads and Illumina 100-bp paired-end reads. In brief, genomic DNA (gDNA) was prepared using the Qiagen Genomic-Tip Kit (20/G) from overnight cultures of *B. cenocepacia* IST439 grown in LB at 37ºC using manufacturer’s instructions. Importantly, this kit uses gravity filtration to purify gDNA, which limits shearing and increases the average fragment size of the resulting gDNA sample. Long insert library preparation and SMRT sequencing was performed on IST439 gDNA at the Icahn School of Medicine at Mount Sinai according to the manufacturer’s instructions, as described previously (Beaulaurier et al., 2015). Briefly, libraries were size selected using Sage Science Blue Pippin 0.75% agarose cassettes to enrich for long-reads, and were assessed for quantity and insert size using an Agilent DNA 12,000 gel chip. Primers, polymerases, and magnetic beads were loaded to generate a completed SMRTbell library, which was run in a single SMRT cell of a Pacific Biosciences RSII sequencer at a concentration of 75 pM for 180 minutes. We used the hierarchical genome-assembly process workflow (HGAP3) to generate a completed assembly of *B. cenocepacia* IST439 and polished our assembly using the Quiver algorithm (Chin et al., 2013).

### Variant calling and SNP/INDEL detections

Trimmed paired-end reads were mapped against the reference complete genome of *B. cenocepacia* IST439 (for all *B. cenocepacia* clonal variants) and against the reference draft genome sequence of *B. multivorans* IST419 for its clonal variants using BWA-MEM packages of Burrows-Wheeler Aligner (BWA v.0.7.10) (Li and Durbin, 2010) and NovoAlign ([www.novocraft.com](http://www.novocraft.com)). Variants [Single nucleotide polymorphisms (SNPs) and insertion-deletion mutations (INDELs)] were called as described previously by using two independents, commonly used, standard variant calling pipelines; GATKand SAMtools/BCFtools toolbox (Li et al., 2009; Van der Auwera et al., 2013; Dillon et al., 2015; Dillon et al., 2017). Briefly, we used SAMtools to convert the SAM alignment files produced for each clonal isolate to mpileup format (Li et al., 2009), then in-house perl scripts were used to produce the forward and reverse read alignments for each position in each line. Next, a three-step process was used to detect putative polymorphisms. First, a base for each individual isolate was called if a site was covered by at least two forward and two reverse reads, and at least 80% of those reads identified the same base. Otherwise, the site was not analysed. Second, an ancestral consensus was called as the base with the highest support among reads across all clonal isolates, as long as there were at least three isolates with sufficient coverage to identify a base. Lastly, at sites where both an individual line base and ancestral consensus were identified, individual line bases were compared to the ancestral base, and if they were different, a putative base-substitution mutation was identified. This analysis was carried out independently with the alignments generated by BWA and Novoalign, and putative SNPs were considered genuine only if both pipelines independently identified the mutation. Finally, the called variants of both pipelines (the above in-house perl scripts and GATK) were combined and filtered with the SAMtools/BCFtools toolbox, v1.9 (Li et al., 2009). In order to avoid false positive calls, maximum read depth (<2000) was considered to avoid any further false duplications and the call confidence was concerned based on RMS mapping quality ≥ 30 and the minimum read depth >5.

All indels identified in this study were also detected using similar requirements to those previously described (Dillon et al., 2015), taking into account that inherent difficulties with gaps and repeat elements can reduce agreement in the alignment of single reads using short-read alignment algorithms, even in the case of true indels. All putative short-indels that were independently identified with the alignments were considered genuine only if both pipelines (the above in-house perl scripts and GATK) independently identified the mutation. To perform a quality control of the above method, a parallel comparison was performed using Breseq(Barrick et al., 2014), and similar predictions were predominantly observed in the case of base-substitutions, with some discrepancies in indels. All putative SNPs/INDELs were then manually inspected and evaluated using the Integrative Genomics Viewer – IGV (Robinson et al., 2011; Thorvaldsdottir et al., 2013), and discarded if the BWA and Novoalign-produced alignments did not provide enough confidence (poor coverage as aformentioned). Thus, we are confident that nearly all base-substitution and indels identified in this study were genuine events that arose during the in-host evolutionary process. Finally, functional annotation of the called variants was performed by using SnpEff v3.1 (Cingolani et al., 2012) with manual BLAST verification against the NCBI Microbes genome database.

Barrick, J.E., Colburn, G., Deatherage, D.E., Traverse, C.C., Strand, M.D., Borges, J.J., et al. (2014). Identifying structural variation in haploid microbial genomes from short-read resequencing data using breseq. *BMC Genomics* 15**,** 1039. doi: 10.1186/1471-2164-15-1039.

Beaulaurier, J., Zhang, X.S., Zhu, S., Sebra, R., Rosenbluh, C., Deikus, G., et al. (2015). Single molecule-level detection and long read-based phasing of epigenetic variations in bacterial methylomes. *Nature Communications* 6**,** 7438. doi: 10.1038/ncomms8438.

Chin, C.S., Alexander, D.H., Marks, P., Klammer, A.A., Drake, J., Heiner, C., et al. (2013). Nonhybrid, finished microbial genome assemblies from long-read SMRT sequencing data. *Nature Methods* 10(6)**,** 563-569. doi: 10.1038/nmeth.2474.

Cingolani, P., Platts, A., Wang le, L., Coon, M., Nguyen, T., Wang, L., et al. (2012). A program for annotating and predicting the effects of single nucleotide polymorphisms, SnpEff: SNPs in the genome of *Drosophila melanogaster* strain w1118; iso-2; iso-3. *Fly (Austin)* 6(2)**,** 80-92. doi: 10.4161/fly.19695.

Dillon, M.M., Sung, W., Lynch, M., and Cooper, V.S. (2015). The Rate and Molecular Spectrum of Spontaneous Mutations in the GC-Rich Multichromosome Genome of *Burkholderia cenocepacia*. *Genetics* 200(3)**,** 935-946. doi: 10.1534/genetics.115.176834.

Dillon, M.M., Sung, W., Sebra, R., Lynch, M., and Cooper, V.S. (2017). Genome-Wide Biases in the Rate and Molecular Spectrum of Spontaneous Mutations in *Vibrio cholerae* and *Vibrio fischeri*. *Molecular Biology and Evolution* 34(1)**,** 93-109. doi: 10.1093/molbev/msw224.

Li, H., and Durbin, R. (2010). Fast and accurate long-read alignment with Burrows-Wheeler transform. *Bioinformatics* 26(5)**,** 589-595. doi: 10.1093/bioinformatics/btp698.

Li, H., Handsaker, B., Wysoker, A., Fennell, T., Ruan, J., Homer, N., et al. (2009). The Sequence Alignment/Map format and SAMtools. *Bioinformatics* 25(16)**,** 2078-2079. doi: 10.1093/bioinformatics/btp352.

Robinson, J.T., Thorvaldsdottir, H., Winckler, W., Guttman, M., Lander, E.S., Getz, G., et al. (2011). Integrative genomics viewer. *Nature Biotechnology* 29(1)**,** 24-26. doi: 10.1038/nbt.1754.

Thorvaldsdottir, H., Robinson, J.T., and Mesirov, J.P. (2013). Integrative Genomics Viewer (IGV): high-performance genomics data visualization and exploration. *Briefings in Bioinformatics* 14(2)**,** 178-192. doi: 10.1093/bib/bbs017.

Van der Auwera, G.A., Carneiro, M.O., Hartl, C., Poplin, R., Del Angel, G., Levy-Moonshine, A., et al. (2013). From FastQ data to high confidence variant calls: the Genome Analysis Toolkit best practices pipeline. *Current Protocols in Bioinformatics* 43**,** 11 10 11-33. doi: 10.1002/0471250953.bi1110s43.
